# Changes in the Vertical Dimension After Orthodontic Treatment in Response to Different Premolar Extraction Patterns

**DOI:** 10.7759/cureus.38893

**Published:** 2023-05-11

**Authors:** Hamad Burashed

**Affiliations:** 1 Orthodontics, St. Barnabas Hospital Health System, Bronx, USA

**Keywords:** cephalometric study, orthodontic treatment, vertical dimension, mandibular plane angle, premolar extractions

## Abstract

Introduction: Premolar extractions in orthodontics can relieve dental crowding and affect incisor inclination. The aim of this retrospective study was to compare changes to the facial vertical dimension after orthodontic treatment with different premolar extraction patterns and non-extraction treatment.

Methods: This was a retrospective cohort study. The pre-and post-treatment records of patients with at least 5.0mm of dental arch crowding were accessed. Patients were divided into three groups: Group A, patients who had four first premolars extracted during their orthodontic treatment; Group B, patients who had four second premolars extracted during their orthodontic treatment; and Group C, patients with no extractions during orthodontic treatment. The pre-and post-treatment skeletal vertical dimension measured through the mandibular plane angle, as well as the incisor angulations/positions, were measured on lateral cephalograms and compared between groups. Descriptive statistics were computed and statistical significance was set at p<0.05. A one-way ANOVA test was conducted between groups to assess for statistically significant differences in changes to the mandibular plane angle and incisor positions/angulations. Post-hoc statistical analyses were performed between groups for parameters showing significant differences.

Results: One-hundred twenty-one patients were included (47 males and 74 females) with ages ranging from 9 years old to 26 years old. Mean upper dental crowding ranged from 6.0 to 7.3mm and mean lower crowding ranged from 5.9-7.4mm across groups. There were no significant differences in mean age, mean treatment length, or mean dental arch crowding in each arch across groups. There were no significant differences in changes to the mandibular plane angle across all three groups regardless of the extraction pattern or non-extraction during orthodontic treatment. The upper and lower incisors were significantly retracted in groups A and B and significantly protracted in group C post-treatment. The upper incisors retroclined significantly more in Group A than Group B and proclined significantly in Group C.

Conclusion: No differences in the vertical dimension and the mandibular plane angle were observed when extracting first versus second premolars and in non-extraction treatment. Significant changes to the incisor inclinations/position were observed depending on the extraction/non-extraction pattern executed. Different premolar extraction patterns during orthodontic treatment do not influence changes to the vertical dimension. Clinicians should make extraction decisions based on desired outcomes for incisors rather than controlling the vertical dimension.

## Introduction

Premolar extraction decisions in orthodontic treatment are often made to relieve dental arch crowding. Several other factors may also weigh into clinicians’ decisions to extract premolars, including the need to retract incisors or the need for soft tissue profile reduction [1-7]. One of the many factors that influence orthodontists’ decisions to extract premolars may also include a desire to control or influence the vertical facial dimension and mandibular plane angle [8]. The vertical skeletal and/or facial dimension in orthodontics is often measured and represented by the mandibular plane angle defined as the angle of the mandibular body relative to the cranial base. The mandibular plane angle can be measured on a lateral cephalometric radiograph through two parameters: SN-MP (angle between sella-nasion to the mandibular plane) and FMA (angle between the Frankfort horizontal plane (porion-orbitale) and the mandibular plane).

Several studies have shown that extracting premolars can help control, or sometimes reduce, the vertical dimension through a decrease in the mandibular plane angle [9-12]. The concept of the “wedge effect” is predicated on the belief that when premolars are extracted, posterior teeth may be able to move mesially, reducing the “wedge effect” and either controlling or reducing the vertical skeletal height or mandibular plane angle [11-14].

Some previous studies have supported the concept that premolar extractions can reduce vertical facial height [11,12]. Sassouni and Nanda found that premolar extractions led to a reduction in facial height when compared to non-extraction treatment [15]. Another study by Garlington and Logan showed that enucleating second premolars leads to a reduction in the mandibular plane angle [16].

Other studies, however, have disproved the influence of premolar extractions on the mandibular plane angle and vertical facial dimension [17-20]. Although these findings exist in the literature, all these previous studies investigated the influence of first premolar extractions on the mandibular plane angle when compared to non-extraction treatment. There are few to no studies that compared differences between first premolar versus second premolar extractions in changes to the vertical dimension/mandibular plane angle. If the theory of the “wedge effect” holds true, it is reasonable to hypothesize that extracting second premolars, which is closer to the “wedge”, may lead to a greater decrease in the vertical dimension and mandibular plane angle post-treatment than extracting the first premolars.

The aim of this study was to compare changes to the vertical dimension/mandibular plane angle when extracting first premolars versus second premolars in patients presenting with moderate-severe dental arch crowding. By assessing the effects of different premolar extraction patterns on the mandibular plane angle, clinicians can make better decisions on which premolars to extract if the mandibular plane angle needs to be controlled or influenced during the course of orthodontic treatment.

The specific aims of this study were: (1) to examine if there was a change to the mandibular plane angle when extracting first premolars, extracting second premolars, or when performing the non-extraction treatment, and (2) to assess if the mandibular plane angle changes were greater when extracting first premolars versus second premolars.

## Materials and methods

This was a retrospective cohort study. This study received ethical approval from the St. Barnabas Hospital Institutional Review Board (IRB approval #: 2018.58), and all patients included had appropriately given their informed consent/assent. Patients were identified for inclusion in this study by reviewing the pre-treatment and post-treatment orthodontic records of all patients who completed comprehensive orthodontic treatment in the St. Barnabas Hospital orthodontic dental clinic and two private practices. Table 1 outlines the inclusion and exclusion criteria for the study.

**Table 1:** Inclusion and Exclusion Criteria of the Study

Inclusion Criteria	Exclusion Criteria
Comprehensive orthodontic treatment.	Limited or Interceptive orthodontic treatment
Patients treated with fixed labial orthodontic appliances	Patients in early mixed dentition
Pre-and post-treatment cephalograms are available	Asymmetric premolar extractions within a dental arch
Pre-and post-treatment intraoral and extraoral photographs are available.	Less than four premolars were extracted.
Pre-treatment crowding greater than or equal to 5mm	More than four premolars were extracted.
Initial Angle’s Class 1 molar classification bilaterally.	Different premolars extracted in different arches (eg. class II or class III extraction pattern)
Moderate anchorage in extraction cases with at least 2.0mm of mesial molar movement in all quadrants.	Other permanent teeth extracted other than premolars (excluding third molar extractions)
Initial ANB of 0 to 5 degrees indicates a skeletal Class 1 pattern.	Patients with craniofacial anomalies
Y-axis ranging from 52 to 66 degrees indicates a normodivergent vertical growth pattern.	Patients with syndromes
	Patients who had orthognathic surgery
	Patients with congenitally missing teeth (other than third molars)
	Patients with supernumerary teeth.
	Patients presenting with severe Bolton discrepancies.
	Patients who used extraoral appliances (such as headgear) or intraoral appliances (fixed class II correctors, temporary anchorage devices, etc).

Patients were included in the study if they presented with a skeletal class 1 presentation (ANB values ranging from 0 to 5 degrees), Angle’s class 1 molar relationship, and normodivergent vertical growth pattern with Y-axis values ranging from 52 degrees to 66 degrees, and had comprehensive non-surgical orthodontic treatment with fixed labial orthodontic appliances (braces) involving both dental arches. All patients included in the study had pre-treatment and post-treatment lateral cephalometric radiographs. All included patients were verified to exhibit class 1 skeletal and class 1 dental relationships as well as normodivergent vertical growth patterns as per their ANB and Y-axis values as well as Angle's molar classifications. Patients were divided into three treatment groups, Groups A, B, and C. Group A included patients with moderate-severe dental arch crowding (defined as crowding greater than or equal to 5.0mm in both arches) who had four first premolars extracted during orthodontic treatment. Group B included patients with moderate-severe dental arch crowding who had four second premolars extracted during orthodontic treatment. Group C included patients with moderate-severe dental arch crowding who had no permanent teeth extracted during orthodontic treatment. All patients in extraction groups A and B were treated with moderate anchorage protocols in which space closure after premolars were extracted involved at least 2.0mm of mesial molar movement into the extraction spaces in all quadrants.

**Figure 1:**
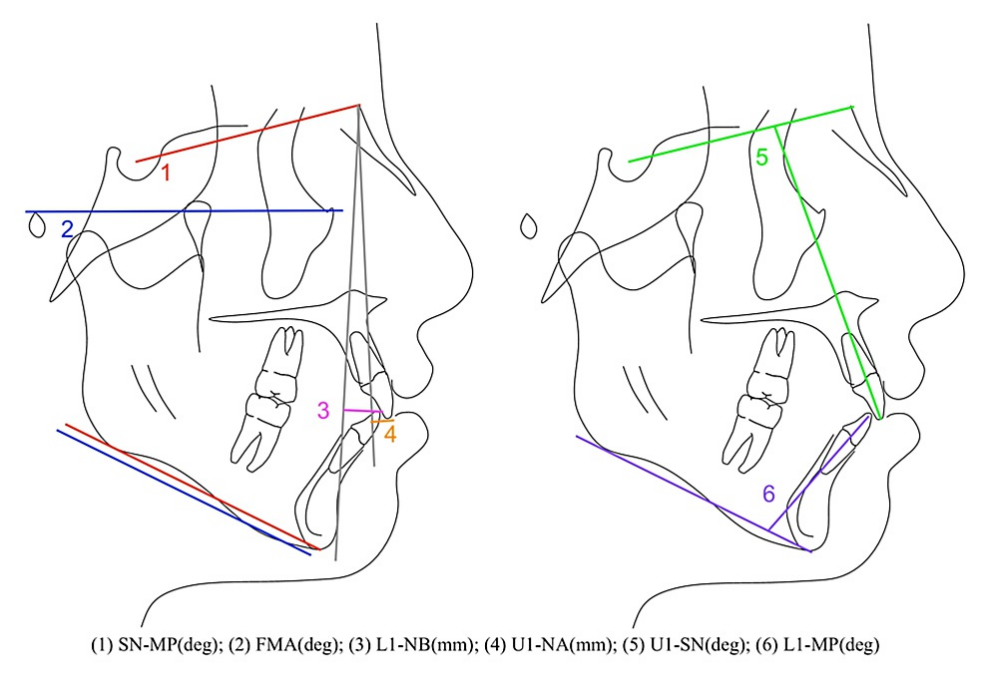
Cephalometric Parameters Analyzed at T0 and T1 *original image created by the author of this investigation (1) SN-MP = angle of the mandibular plane to sella-nasion; this represents the mandibular plane angle and vertical dimension (2) FMA = "Frankfort Mandibular Angle": angle of the mandibular plane to the Frankfort Horizontal Plane represented by porion-orbitale; this also represents the mandibular plane angle and vertical dimension (3) L1-NB = anterior-posterior position of the lower central incisor incisal edge relative to the vertical line connecting nasion-B point (NB); this represents the spatial position of the lower incisor relative to the cranial base (4) U1-NA = anterior-posterior position of the upper central incisor incisal edge relative to the vertical line connecting nasion-A point (NA); this represents the spatial position of the upper incisor relative to the cranial base (5) U1-SN = angulation of the upper central incisor relative to sella-nasion; angle of the central incisor relative to the cranial base (6) L1-MP = angulation of the lower central incisor relative to the mandibular plane

All patients had pre-treatment intraoral scans or digital scans of their plaster models. The pre-treatment dental arch crowding for each patient was measured digitally by subtracting the total mesiodistal widths of the permanent teeth from the corresponding arch perimeter using OrthoCAD software (Cadent, Inc., Carlstadt, NJ). The mean upper arch crowding and mean lower arch crowding was calculated for each group. The sex, pre-treatment age, and total treatment length of each patient were recorded. Pre-treatment (T0) and post-treatment (T1) lateral cephalograms were traced using Dolphin imaging software (Patterson Dental, Chatsworth, CA) and assessed by the same investigator at two different time points to ensure adequate intrarater reliability. The hard tissue and dental cephalometric parameters recorded at T0 and T1 are outlined in Figure 1 and listed below.

The following parameters were measured and recorded for each patient in all groups at T0 and T1, and the means and standard deviations of these measurements were calculated: 1. SN-MP (degrees): The mandibular plane angle relative to the anterior cranial base (sella-nasion). This is one of the measurements used to represent the vertical skeletal dimension. 2. FMA (degrees): The mandibular plane angle relative to the Frankfort horizontal plane (porion-orbitale). This is one of the measurements used to represent the vertical skeletal dimension. 3. U1-SN (degrees): The upper central incisor inclination relative to the anterior cranial base. 4. U1-NA (mm): The upper incisor anteroposterior position relative to the Nasion-A point plane. 5. L1-MP (degrees): The lower incisor inclination relative to the mandibular plane. 6. L1-NB (mm): The lower incisor anteroposterior position relative to the Nasion-B point plane.

A power analysis was calculated and a sample size of 28 patients per group was determined to achieve 80% power at an effect size of 0.74. This sample size was derived by using differences in mandibular plane angle changes (SN-MP and FMA). A systematic review by Kouvelis et al. reviewed studies investigating changes to the vertical facial height, including mandibular plane angle, in extraction versus non-extraction treatment [20]. The Kouvelis review found that in studies that showed significant differences in changes to the SN-MP and FMA between extraction vs. non-extraction cohorts, a 1.7 degree difference in SN-MP changes and a 2.3 degree difference in FMA changes with a 2.3 degree standard deviation for both parameters was found as statistically significant in two investigations within the review. Utilizing these values, an effect size of 0.74 and 1.0 were computed, respectively. Using the smaller effect size of 0.74, a sample size of 28 patients per group was calculated for this study to achieve 80% power at a 95% confidence interval.

Statistical analysis

Descriptive statistics were computed and statistical significance was set at p<0.05. The mean values for each cephalometric parameter at T0 and T1, and the mean change from T0 to T1, were calculated. Intraclass correlation coefficients were calculated to assess intra-rater reliability. The mean cephalometric values at T0 and T1 were compared to population normative values using the z-test. Population norm values for all measured parameters are outlined in Table 2.

**Table 2:** Population Norm Values of Cephalometric Landmarks Landmark Definitions: SN-MP (degrees): The mandibular plane angle relative to the anterior cranial base (sella-nasion). This is one of the measurements used to represent the vertical skeletal dimension. FMA (degrees): The mandibular plane angle relative to the Frankfort horizontal plane (porion-orbitale). This is one of the measurements used to represent the vertical skeletal dimension. U1-SN (degrees): The upper central incisor inclination relative to the anterior cranial base. U1-NA (mm): The upper incisor anteroposterior position relative to the Nasion-A point plane. L1-MP (degrees): The lower incisor inclination relative to the mandibular plane. L1-NB (mm): The lower incisor anteroposterior position relative to the Nasion-B point plane.

Cephalometric Landmark	Norm Value
SN-MP	36.0 degrees
FMA (FH-MP)	25.0 degrees
U1-NA	4.3 mm
U1-SN	103.6 degrees
L1-NB	4.0 mm
L1-MP	96.0 degrees

A paired t-test was calculated from T0 to T1 for each parameter within each group to assess if there was a statistically significant change pre- to post-treatment for each value. One-way ANOVA was performed for each parameter across groups at both T0 and T1 as well as for T0-T1 changes across groups to assess for statistically significant differences. Post-hoc Tukey tests were performed between groups for parameters showing statistically significant differences. Statistical analyses were performed using Microsoft Excel v16.0 software.

## Results

Sample characteristics

The records of 153 patients who met the inclusion criteria were reviewed. Of these patients, 32 patients were eliminated due to meeting one of the exclusion criteria or due to lack of high-quality records. The final sample size had 121 patients divided between Groups A to C. The majority of the patients were of Latino/Latina origin.

The demographic characteristics of the sample in each group and the starting pre-treatment values are outlined in Table 3. Fifty patients were included in Group A (first premolar extraction group), 31 patients were included in Group B (second premolar extraction group), and 40 patients were in Group C (non-extraction group). A one-way analysis of variance (ANOVA) revealed that there were no significant differences in age between groups at the start of treatment (p>0.05) and no difference in treatment length across groups (p>0.05). Significant differences in both mean upper and mean lower dental arch crowding were revealed through the one-way analysis of variance (p<0.05). A post hoc Tukey test revealed significant differences in both upper arch (p<0.05) and lower arch crowding (p<0.05), but there were no statistically significant differences in initial upper and lower dental arch crowding between the two extraction groups A and B (p>0.05). There was significantly larger upper and lower crowding in Group A when compared to non-extraction Group C (p<0.05) as revealed by the post hoc Tukey test.

**Table 3:** Demographic Pre-Treatment Data (T0) and Statistical Significant Differences of T0 Values Between Groups A to C *  Z-test performed; FMA at T0 is statistically significantly larger than normal (FMA = 25) for Groups A, B, C ** Z-test performed; U1-NA and U1-SN at T0 each statistically significantly larger than normal for Group A *** Z-test performed; L1-NB at T0 is statistically significantly larger than normal (4.0mm) for Groups A, B, C **** one-way ANOVA performed for each parameter across groups; reported groups indicate statistically significant difference at T0 between each pair of reported groups at p < 0.05 as revealed by a post-hoc Tukey test SN-MP (degrees): The mandibular plane angle relative to the anterior cranial base (sella-nasion). This is one of the measurements used to represent the vertical skeletal dimension. FMA (degrees): The mandibular plane angle relative to the Frankfort horizontal plane (porion-orbitale). This is one of the measurements used to represent the vertical skeletal dimension. U1-SN (degrees): The upper central incisor inclination relative to the anterior cranial base. U1-NA (mm): The upper incisor anteroposterior position relative to the Nasion-A point plane. L1-MP (degrees): The lower incisor inclination relative to the mandibular plane. L1-NB (mm): The lower incisor anteroposterior position relative to the Nasion-B point plane. # : number

	GROUP A (N = 50)	GROUP B (N = 31)	GROUP C (N = 40)	Inter-Group Statistically Significant Differences ****
Mean Age (years)	13.0 + 2.9	13.2 + 5.1	13.7 + 3.6	-
Mean Tx Length (months)	30.6 + 5.8	35.8 + 14.6	31.2 + 9.5	-
# Males : # Females	23 : 27	9 : 22	15 : 25	-
Mean Upper Crowding (mm)	7.3 + 3.1	6.6 + 2.6	6.0 + 2.0	A|C
Mean Lower Crowding (mm)	7.4 + 2.5	6.5 + 2.3	5.9 + 2.0	A|C
SN-MP (˚)	36.8 + 6.0	35.7 + 5.8	34.5 + 5.2	-
FMA (FH-MP) (˚)	29.4 + 5.0*	28.2 + 3.8*	27.7 + 3.8*	-
U1-NA (mm)	6.0 + 2.4**	5.1 + 2.6	3.9 + 3.3	A|C B|C
U1-SN (˚)	109.2 + 7.1**	105.7 + 8.9	103.4 + 10.1	A|C
L1-NB (mm)	8.5 + 2.9***	7.4 + 3.5***	6.0 + 2.8***	A|C
L1-MP (˚)	94.1 + 7.7	95.0 + 7.3	91.3 + 5.4	A|C B|C

A subsample of each group’s T0 and T1 cephalograms was re-traced by the same examiner and the SN-MP, FMA, and incisor position/inclination parameters were re-measured. Intraclass correlation coefficients showed excellent intra-rater reliability ranging from ICC=0.912-0.989.

Pre-treatment cephalometric values (T0) for vertical dimension (mandibular plane angle) and incisor positions

Table 3 above outlines the initial cephalometric values at T0 related to incisor position, incisor angulation, and the vertical dimension/mandibular plane angle in each group. One-way analysis of variance (ANOVA) revealed no statistically significant differences in the pre-treatment vertical dimension/mandibular plane angle represented by both SN-MP and FMA across all groups (p>0.05). This shows that all treatment groups began with similar vertical dimension/mandibular plane angle presentations. Initial SN-MP was not significantly different than normal across all groups (p>0.05). However, all groups showed higher FMA values than normal at T0 (p<0.05).

One-way ANOVA tests showed statistically significant differences between groups A to C for initial U1-NA position, U1-SN angulation, L1-NB position, and L1-MP angulation (p<0.05). U1-NA and U1-SN were both significantly larger than normal for group A (p<0.05). L1-NB was significantly larger than normal across all groups at T0 (p<0.05). Post-hoc Tukey tests for initial T0 values showed that extraction groups A and B had greater initial U1-NA and L1-MP values than non-extraction Group C (p<0.05). Post-hoc Tukey tests also showed that group A had significantly larger initial U1-SN and L1-NB than group C (p<0.05).

Post-treatment cephalometric values (T1) for vertical dimension (mandibular plane angle) and incisor positions

Table 4 shows the post-treatment cephalometric T1 values for each group. One way analysis of variance (ANOVA) revealed no statistically significant differences in the final vertical dimension/mandibular plane angle values for both SN-MP and FMA between any of the groups (p>0.05).

**Table 4:** Cephalometric Values at T1 and Statistical Significant Differences of T1 Values Between Groups A to C * Z-test performed; Indicates statistically significant difference from norm values at p < 0.05 ** one-way ANOVA performed for each parameter across groups; reported groups indicate statistically significant difference at T1 between each pair of reported groups at p < 0.05 as revealed by a post-hoc Tukey test SN-MP (degrees): The mandibular plane angle relative to the anterior cranial base (sella-nasion). This is one of the measurements used to represent the vertical skeletal dimension. FMA (degrees): The mandibular plane angle relative to the Frankfort horizontal plane (porion-orbitale). This is one of the measurements used to represent the vertical skeletal dimension. U1-SN (degrees): The upper central incisor inclination relative to the anterior cranial base. U1-NA (mm): The upper incisor anteroposterior position relative to the Nasion-A point plane. L1-MP (degrees): The lower incisor inclination relative to the mandibular plane. L1-NB (mm): The lower incisor anteroposterior position relative to the Nasion-B point plane.

	GROUP A	GROUP B	GROUP C	Inter-Group Statistically Significant Differences **
SN-MP (˚)	36.2 + 5.4	35.3 + 5.3	34.0 + 5.7	-
FMA (FH-MP) (˚)	28.9 + 4.7*	27.4 + 3.8*	27.1 + 4.1*	-
U1-NA (mm)	3.3 + 2.8	3.5 + 3.2	5.0 + 2.3*	A|C
U1-SN (˚)	102.7 + 6.3	104.4 + 9.5	108.1 + 8.3*	A|C
L1-NB (mm)	6.8 + 2.5*	5.5 + 2.8*	8.1 + 2.4*	A|B A|C B|C
L1-MP (˚)	92.0 + 7.6	90.4 + 8.4	97.5 + 6.6	A|C B|C

One-way ANOVA tests did reveal significant variations at T1 for U1-NA, U1-SN, L1-NB, and L1-MP. Post-hoc Tukey tests showed the following results. Both the final U1-NA and U1-SN were significantly smaller in Group A than in Group C (p<0.05). Both extractions groups A and B had significantly smaller L1-MP inclinations than group C post-treatment (p<0.05). Final L1-NB was significantly smaller in both groups A and B than in group C (p<0.05). Group A showed a significantly larger final L1-NB than Group B at T1 (p<0.05).

Table 4 also outlines the comparisons of the values at T1 to population norm values. At T1, FMA remained significantly larger than normal post-treatment (p<0.05) across all groups. Final U1-NA and final U1-SN angulation were significantly larger than normal for non-extraction group C (p<0.05). The final L1-NB incisor position was significantly larger than normal post-treatment in all groups (p<0.05).

Post-treatment changes in the vertical dimension (mandibular plane angle) and incisor positions (T0 to T1)

The post-treatment changes to the vertical dimension/mandibular plane angle and incisor position/angulations in each group are illustrated in Table 5. Paired t-tests showed that there was no statistically significant change in the vertical dimension/mandibular plane angle (SN-MP and FMA) from T0 to T1 in groups A, B, and C (p>0.05). The vertical dimension/mandibular plane angle remained stable with a non-significant change ranging from -0.4 to -0.8 degrees in both extraction groups A and B, as well as in non-extraction group C. One-way analyses of variance (ANOVA) showed that there were also no statistically significant differences in these changes observed for the vertical dimension/mandibular plane angle from T0 to T1 across all three groups (p>0.05).

**Table 5:** Changes in Cephalometric Dimensions from T0 to T1 Within Each Group and Statistical Significant Differences in T0-T1 Changes Between Groups A to C * Paired t-test performed; Indicates statistically significant change from T0 to T1 at p < 0.05 ** one-way ANOVA performed for each parameter across groups; reported groups indicate statistically significant difference for T0-T1 changes between each pair of reported groups at p < 0.05 as revealed by a post-hoc Tukey test SN-MP (degrees): The mandibular plane angle relative to the anterior cranial base (sella-nasion). This is one of the measurements used to represent the vertical skeletal dimension. FMA (degrees): The mandibular plane angle relative to the Frankfort horizontal plane (porion-orbitale). This is one of the measurements used to represent the vertical skeletal dimension. U1-SN (degrees): The upper central incisor inclination relative to the anterior cranial base. U1-NA (mm): The upper incisor anteroposterior position relative to the Nasion-A point plane. L1-MP (degrees): The lower incisor inclination relative to the mandibular plane. L1-NB (mm): The lower incisor anteroposterior position relative to the Nasion-B point plane.

	GROUP A	GROUP B	GROUP C	Inter-Group Statistically Significant Differences **
SN-MP (˚)	-0.6 + 2.6	-0.4 + 2.5	-0.6 + 2.1	-
FMA (FH-MP) (˚)	-0.5 + 1.7	-0.8 + 2.8	-0.6 + 1.9	-
U1-NA (mm)	-2.6 + 2.9*	-1.5 + 2.5*	1.1 + 3.1*	A|C B|C
U1-SN (˚)	-6.6 + 6.5*	-1.3 + 7.0	4.8 + 10.8*	A|B A|C B|C
L1-NB (mm)	-1.8 + 2.6*	-1.9 + 2.2*	2.2 + 2.0*	A|C B|C
L1-MP (˚)	-2.1 + 8.6	-4.6 + 6.8*	6.2 + 6.3*	A|C B|C

Paired t-tests revealed that statistically significant changes from T0 to T1 were observed for the upper and lower incisor positions/angulations. The upper incisors (U1-NA) significantly retracted from T0 to T1 for extraction groups A and B and protracted forward in non-extraction group C (p<0.05). The upper incisor angulation (U1-SN) retroclined significantly in Group A and proclined significantly in Group C from T0 to T1 (p<0.05). The lower incisors (L1-NB) retracted significantly in extraction Groups A and B, and significantly protracted forward in non-extraction Group C from T0 to T1 (p<0.05). The lower incisor inclination (L1-MP) retroclined significantly in Group B and proclined significantly in non-extraction Group C from T0 to T1 (p<0.05). When these changes were compared between groups, one-way ANOVA analyses revealed statistically significant differences in the post-treatment changes (T0-T1) which are outlined in Table 5. Of particular note is changes seen to U1-SN between Groups A and B. A post hoc Tukey test revealed a significantly greater amount of U1-SN retroclination in group A than in group B (p<0.05).

## Discussion

Premolars are often extracted during orthodontic treatment to alleviate dental crowding. In borderline cases with moderate-to-severe dental crowding where the decision to extract is ambiguous, additional factors may drive orthodontists to decide if and which premolars to extract. Clinical presentation and desired outcomes related to planned final incisor positions, incisor angulations, soft tissue profile, and vertical facial height play significant roles in clinicians’ decisions to follow a certain extraction or non-extraction protocol.

The vertical skeletal component (represented by the mandibular plane angle) is an important consideration for clinicians when planning extraction-based or non-extraction orthodontic treatment. In cases with crowding where the vertical dimension/mandibular plane angle needs to be controlled or reduced, some clinicians may favor premolar extraction to help mitigate undesired increases in the vertical facial height. The rationale behind this may be related to the “wedge effect” theory, where premolar extractions may allow mesial movement of the posterior teeth segments, preventing the mandible from clockwise rotation and therefore preventing increases in the vertical dimension and mandibular plane angle [11]. If this hypothesis holds true, conventional wisdom dictates that extracting second premolars versus first premolars may lead to a greater reduction of the vertical dimension (and mandibular plane angle) as second premolar extractions are closer to the “posterior wedge” of patients’ dentition.

The results of this study reject this hypothesis. The findings of this study showed that no differences in changes to the vertical dimension/mandibular plane angle after orthodontic treatment were observed when extracting first premolars, extracting second premolars, or in non-extraction treatment in moderate-severe crowding orthodontic cases. Furthermore, the outcomes of this study revealed that the vertical dimension/mandibular plane angle remained unchanged in all three extraction and non-extraction groups, showing that extraction/non-extraction does not influence changes to the vertical dimension during orthodontic treatment.

Previous studies have suggested that extracting second premolars instead of first premolars during orthodontics may be favorable in crowding cases that present with favorable initial incisor position and angulation [21-25]. In these studies, it was shown that extracting second premolars alleviated dental crowding without significant changes to incisor inclinations. The results of this study had similar findings showing that the first premolar extraction Group A had greater decreases in U1-SN than the second premolar extraction Group B. When it comes to vertical facial height, specifically the mandibular plane angle (SN-MP and FMA), few to no studies made a similar comparison between extracting first versus second premolars.

Many previous studies have compared vertical dimension/mandibular plane angle outcomes related to first premolar extractions versus non-extraction treatment [17-20]. The majority of these studies found that there was no difference in changes to the vertical dimension/mandibular plane angle when extracting first premolars compared to non-extraction. The results of this study add to the existing body of evidence by suggesting that extraction of second premolars also does not lead to significant changes to the vertical dimension/mandibular plane angle when compared to first premolar extractions or non-extraction.

An important consideration in interpreting the findings of this study is the anchorage used during premolar extractions, or how the extraction spaces were managed during orthodontic treatment. If the “wedge effect” theory holds true, the mesial movement of the molars into the premolar extraction spaces would lead to decreases in the vertical dimension values (SN-MP and FMA values) [11]. Therefore, if the anchorage mechanics employed during extraction-based treatment are maximum posterior anchorage (in other words, the entire extraction space was used to distalize the anterior segments into the space), then it would make sense that no changes to the mandibular plane angle would be observed regardless of the extraction pattern. The anchorage mechanics therefore need to allow for mesial movement of the posterior segments into the extraction spaces and these mechanics should be standardized across the extraction groups to make an accurate comparison of differences to SN-MP and FMA. This was indeed considered in this study; all patients who were included in extraction groups A and B had moderate anchorage mechanics in which at least 2.0mm of mesial movement of the molars into the extraction spaces was achieved.

It’s important to realize, however, that although all extraction groups in this study sample had moderate anchorage, the amount of mesial movement of the molars beyond 2.0mm and its correlation to vertical dimension/mandibular plane angle changes was not analyzed or recorded. While extraction of first versus second premolars was analyzed in this study, the ultimate outcome being investigated is if mesial molar movement correlates to vertical dimension/mandibular plane angle changes. Future areas of research should include evaluating how the direction and degree of molar movement into extraction spaces correlate to changes to the vertical dimension/mandibular plane angle. These factors could play an even more significant and meaningful role than analyzing premolar extraction patterns as it relates to mandibular plane angle and vertical dimension.

Another important consideration is the potential vertical growth pattern that patients presented with pre-treatment that could influence the findings of this study. This was indeed considered in this investigation by only including patients whose Y-axis values represented a normodivergent growth pattern that was not excessively vertical or horizontal in future growth potential. While this was considered, the difference in age and potential maturity levels across patients between groups could add the confounding variability of patients being at different stages of growth and development that could pose a greater influence on the vertical dimension observed than the premolar extractions that were done. Future studies can standardize the sample of patients by only including those patients who not only exhibited similar Y-axis values but who also had hand-wrist radiographs and were at the same SMI maturity level and/or CVMS maturity level.

Although all groups in this study met the minimum sample size to achieve adequate statistical power, this study still had a relatively small sample size. With a greater sample size, one would be able to stratify the data and consider further factors including anchorage considerations, size of extraction spaces, periodontal considerations, tissue biotype, and other factors that could influence the results of this study.

Due to the retrospective nature of this study, it was difficult to standardize the treatment mechanics across the treatment groups. The non-uniformity of the biomechanics used including the use of intermaxillary elastics and other biomechanics that may extrude or intrude posterior segments can certainly introduce confounding variables that can influence the vertical dimension. Furthermore, the growth pattern including the skeletal maturity stage of each patient which may influence findings, as well as the type of anchorage in all three dimensions, along with persistent habits may all have additional effects that may compound the findings of this study.

## Conclusions

The results of this retrospective study show that the vertical dimension (represented by the mandibular plane angle) remains stable post-orthodontic treatment in both premolar extraction and non-extraction treatment. Extracting first premolars versus second premolars during orthodontic treatment does not influence changes observed to the mandibular plane angle or vertical facial height.

Clinicians’ decisions on premolar extraction patterns or non-extraction treatment during orthodontic treatment should not be influenced by desired control or changes to the vertical dimension. Clinicians should base their orthodontic extraction decisions on other factors, including crowding and desired changes to the incisor positions.

Future studies analyzing the correlation of mesial molar movement and anchorage loss to the vertical facial height may provide a better understanding of the influences of premolar extractions on the mandibular plane angle. The amount of anchorage loss may possibly play a greater role in the vertical dimension than the location of premolar extractions.
